# The Lords' Committee

**Published:** 1891-07-25

**Authors:** 


### The Lords' Committee.

The final meeting for the purpose of taking evidence was held on the 13th inst. The members of the Committee present were Lord Sandhurst (Chairman), the Earl of Kimberley, Earl Spencer, Earl Cathcart, Lord Clifford of Chudleigh, and Lord Thring.

Mr. Rathbone, M.P., examined by the Chairman, said he had been for thirty years a member of the Executive Committee of the Liverpool Infirmary, President of the Liverpool Training School for Nurses, a member of the Liverpool Board of Guardians, and a trustee of the Nightingale School for Nurses. The condition of hospitals had very much improved during the last 30 years. Thirty years ago they were in a wretched state, and there were only two hospitals in England that had a proper system of training for nurses—St. Thomas's and King's College. He complained that while there was ample opportunity now for training nurses, there was no means for training Superintendents. The plan pursued in the Liverpool Infirmary to insure effective management was for the Committee to elect every fortnight a member whose duty it should be to visit for a month, so that there were always two visitors, thus giving a certain continuity to the visiting. The medical staff were entirely responsible for the medical treatment of their patients, but the engagement, dismissal, and management of the nurses rested entirely with the Matron. The Committee, however, had power to consider appeals to them. The power which was given to the Matron was not to his knowledge abused, as in the management of their hospitals he had never had a case of wrongful dismissal under his notice.

The Rev. N. Bromley, Warden of King's College Hospital, then gave evidence in correction of some of the statistics which had been placed before the Committee by Mr. Henry C. Burdett in connection with this hospital. For the last three years the expense per occupied bed of King's College Hospital—the average number of occupied beds being 169—was £93 16s., and not £120 14s. 10d. as had been stated. The mistake arose through the exceptional year, 1887, having been included. In that year they had spent a legacy of £9,000 in enlarging the hospital and adding a floor for the accommodation of the nurses. This was quite unusual, and no other term of three years would give so large an amount.

The Committee then adjourned, and will devote the further sittings to the consideration of their report.

